# Targeted metabolomic profiling of acute ST-segment elevation myocardial infarction

**DOI:** 10.1038/s41598-024-75635-3

**Published:** 2024-10-11

**Authors:** Sergey S. Markin, E. A. Ponomarenko, Yu. A Romashova, T. O. Pleshakova, S. V. Ivanov, V. V. Beregovykh, S. L. Konstantinov, G. I. Stryabkova, Zh. Yu. Chefranova, Y. A. Lykov, I. M. Karamova, A. G. Koledinskii, K. M. Shestakova, P. A. Markin, N. E. Moskaleva, S. A. Appolonova

**Affiliations:** 1 Institute of Biomedical Chemistry, Moscow, 119121 Russia; 2 Belgorod Regional Clinical Hospital of St. Joseph, Belgorod, 308007 Russia; 3 Belgorod State National Research University, Belgorod, 308015 Russia; 4 Ufa Emergency City Clinical Hospital, Ufa, 450092 Russia; 5 Peoples’ Friendship University of Russia, Moscow, 117198 Russia; 6 Laboratory of Pharmacokinetics and Metabolomic Analysis, Institute of Translational Medicine and Biotechnology, I.M. Sechenov First Moscow State Medical University (Sechenov University), Moscow, 119435 Russia; 7 I.M. Sechenov First Moscow State Medical University, (Sechenov University), Moscow, 119435 Russia

**Keywords:** Metabolomic profiling, Metabolic pathways, STEMI pathogenesis, Biochemistry, Molecular biology, Biomarkers, Risk factors

## Abstract

Myocardial infarction is a major cause of morbidity and mortality worldwide. Metabolomic investigations may be useful for understanding the pathogenesis of ST-segment elevation myocardial infarction (STEMI). STEMI patients were comprehensively examined via targeted metabolomic profiling, machine learning and weighted correlation network analysis. A total of 195 subjects, including 68 STEMI patients, 84 patients with stable angina pectoris (SAP) and 43 non-CVD patients, were enrolled in the study. Metabolomic profiling involving the quantitative analysis of 87 endogenous metabolites in plasma was conducted. This study is the first to perform targeted metabolomic profiling in patients with STEMI. We identified 36 significantly altered metabolites in STEMI patients. Increased levels of four amino acids, eight acylcarnitines, six metabolites of the NO–urea cycle and neurotransmitters, and three intermediates of tryptophan metabolism were detected. The following metabolites exhibited decreased levels: six amino acids, three acylcarnitines, three components of the NO–urea cycle and neurotransmitters, and three intermediates of tryptophan metabolism. We found that the significant changes in tryptophan metabolism observed in STEMI patients—the increase in anthranilic acid and tryptophol and decrease in xanthurenic acid and 3-OH-kynurenine—may play important roles in STEMI pathogenesis. On the basis of the differences in the constructed weighted correlation networks, new significant metabolite ratios were identified. Among the 22 significantly altered metabolite ratios identified, 13 were between STEMI patients and non-CVD patients, and 17 were between STEMI patients and SAP patients. Seven of these ratios were common to both comparisons (STEMI patients vs. non-CVD patients and STEMI patients vs. SAP patients). Additionally, two ratios were consistently observed among the STEMI, SAP and non-CVD groups (anthranilic acid: aspartic acid and GSG (glutamine: serine + glycine)). These findings provide new insight into the diagnosis and pathogenesis of STEMI.

**Supplementary Information:**

The online version contains supplementary material available at 10.1038/s41598-024-75635-3.

## Introduction

Myocardial infarction (MI) is a major cause of morbidity and mortality worldwide. The Third Universal Definition of Myocardial Infarction declares that any necrosis due to prolonged myocardial ischaemia should be classified as myocardial infarction^1^. Among the five types of MI, spontaneous myocardial infarction (MI type 1) is related to the rupture of atherosclerotic plaques with intraluminal thrombi in coronary arteries. This leads to obstruction of the arteries, decreased myocardial blood flow and myocardial necrosis. MI type 1 is divided into two subtypes: ST-segment elevated MI (STEMI) and non-ST-segment elevated MI (non-STEMI). Patients with both MI 1 subtypes (STEMI and non-STEMI) have clinical features of acute coronary syndrome (ACS) of atherothrombotic origin and increased biomarkers. The STEMI and non-STEMI patients may have a history of angina pectoris of different functional classes, but 5–20%, mainly women, may not have had a history of nonobstructive coronary artery disease on coronary angiography. Patients with acute coronary syndrome without ST-segment elevation and without an increase in biomarkers are classified as having unstable angina of thrombosis-mediated origin^2^.

The clinical diagnosis of STEMI is mainly based on the clinical symptoms of the patient, electrocardiogram, coronary angiography, nuclear magnetic resonance with gadolinium contrast, myocardial necrosis markers, and other auxiliary examinations. These diagnostic strategies are effective but do not provide insight into STEMI because late gadolinium enhancement results in a grey zone consisting of ischaemic and live myocardium in the early stage of STEMI. Recent research has suggested that casitas B lymphoma-b (Cbl-b), an E3 ubiquitin ligase, may serve as a potential biomarker for myocardial infarction, providing evidence that Cbl-b expression is significantly lower in MI patients than in control individuals, as demonstrated by analyses of both a public database and 20 peripheral blood samples^3^. However, this marker is not specific for STEMI. Therefore, clinical biomarkers (such as troponin and creatine kinase), which are widely used for STEMI diagnosis, can only be assessed 4–6 h after the onset of the disease and only discriminate a zone of necrosis^4^, and magnetic resonance with gadolinium contrast is a rarely used method in routine clinical practice. Thus, new approaches are needed for the early diagnosis of myocardial damage in patients with STEMI.

In recent decades, the active growth of omics technologies, particularly metabolomics, has increased their utilization in the clinic for the diagnosis of various disorders, including cardiovascular diseases.

Metabolomics enables the comprehensive characterization of small-weight molecules, such as carbohydrates, amino acids, lipids, nucleotides, and peptides, providing a snapshot of an individual’s metabolic state at a particular time^5^. Unlike other omics technologies, metabolomics integrates genetic, transcriptomic and proteomic data, as well as nutritional and environmental factors, which are included in the diagnostic platform^6^.

Metabolite research can reflect the physiological or pathological state of an organism^7^. The targeted metabolomic profiling utilized in the present study is based on the absolute quantification of various metabolites related to different chemical and biochemical classes of endogenous metabolites. Compared with nontargeted metabolomic analysis, this method provides high levels of sensitivity and selectivity^7^.

The aim of the present study was to identify potential biomarkers and new insights into the pathogenesis of STEMI.

## Materials and methods

### Study design

The inclusion and exclusion criteria of the study are presented in Table 1.

**Table 1:** Inclusion and exclusion criteria.

Inclusion Criteria	• Men and women aged 18 years and older.
	• ST-elevation myocardial infarction (STEMI), stable angina pectoris functional class III according to Canadian Cardiovascular Society classification.
	• Availability of signed and dated informed consent of the patient to participate in the study.
Exclusion Criteria	• Myocardial infarction without ST-elevation.
	• Angina pectoris functional class I, II or IV.
	• Type 1 diabetes mellitus.
	• Use of acetaminophen, any vitamins, minerals, amino acids, dietary supplements (including sports drinks and energy drinks), creatinine, alpha-ketoglutarate, malic acid, citric acid, maleic acid, and orotic acid in the 4 days before blood sampling. Consumption of sweeteners (aspartame, among others), monosodium glutamate or alcohol 24 h before blood sampling (non-CVD group and SAP group).*
	• Any other diseases or conditions that, in the opinion of the investigator, may distort the results of the study and limit the patient’s participation in the study.

*Patients with STEMI who violated their diet or consumed alcohol were excluded from the data analysis after blood sampling.

### Ethical considerations

All experiments were approved by the Ethics Committee of Belgorod Regional Clinical Hospital of St. Joseph, Belgorod, Russia (protocol no. 10, 16 November 2015), in accordance with the ethical principles for medical research involving humans as stated in the Declaration of Helsinki. Written informed consent was signed by all the participants before the beginning of the study.

### Baseline characteristics of the participants

The baseline characteristics of the participants included weight, height, body mass index (BMI), heart rate, blood pressure, and myocardial infarction location.

### Biochemical analysis

The morning after 12 h of overnight fasting, whole blood samples were collected in ethylenediaminetetraacetic acid (EDTA) tubes. Blood samples were immediately centrifuged (2000 rpm, 4 °C) for 20 min to obtain plasma, which was stored at – 80 °C. Biochemical evaluation of the samples included measurements of total cholesterol, triglycerides, HDL-cholesterol, LDL-cholesterol, VLDL-cholesterol, glucose, creatinine, urea, alanine aminotransferase (ALT), aspartate aminotransferase (AST), troponin I, potassium, sodium, calcium, international normalized ratio (INR), activated partial thromboplastin time (APTT) and fibrinogen. Extra plasma aliquots were subject to metabolic analysis at the laboratory of pharmacokinetics and metabolomics.

### Chemicals and reagents

Standard solutions for metabolomic profiling, including methanol, formic acid, and bovine serum albumin (BSA), were obtained from Sigma‒Aldrich (USA). Acetonitrile was purchased from Chromasolv^®^ (Sigma‒Aldrich Chemie GmbH, Buchs, Switzerland). Ultrapure water was obtained from a Millipore Milli‒Q purification system (Millipore Corporation, Billerica, USA). Isotopically labelled standard solutions for metabolic profiling of amino acids and acylcarnitines were obtained from the MassChrom Non Derivatized 57,000 Kit (Chromsystems, Germany), whereas isotope-labelled standard solutions for tryptophan catabolite profiling were obtained from Toronto Research Chemicals (Canada).

### Metabolomic profiling

Targeted metabolomic profiling of the samples was performed in accordance with a previously described method^7^ and included the quantitative analysis of 87 endogenous metabolites in patient plasma. Briefly, sample preparation of amino acids and intermediates of arginine and methionine metabolism consisted of protein precipitation followed by instrumental analysis on a Waters TQ-S-micro triple quadruple mass spectrometer (Waters Corp., Milford, CT, USA). The preparation of samples for acylcarnitine and tryptophan catabolite profiling consisted of liquid‒liquid extraction followed by LC‒MS/MS analysis. The applied methods were validated in accordance with the guidelines for bioanalytical method validation and included assessments of selectivity, linearity, precision, accuracy, recovery, matrix effects, and stability (Supplementary Materials, Tables S1–S8).

### Echocardiography

The left ventricular ejection fraction (LVEF) was assessed using quantitative biplane Simpson measurements.

### Statistical analysis

All the statistical analyses for the characterization of biochemical and metabolic profiles were performed using the Python Stats package^8^. The variable distribution was assessed using the Shapiro‒Wilk test. According to the variable distribution, analysis of variance was performed via the parametric Student’s t test and ANOVA or the nonparametric Kruskal‒Wallis test and Mann‒Whitney U test. After Bonferroni adjustment for multiple comparisons, a p value less than 0.05 was considered to indicate statistical significance. PCA and OPLS-DA were performed using SIMCA-P software^9^. Random forest (RF) analysis was performed via the scikit-learn python package^10^.

### Correlation network analysis

Weighted correlation network analysis was performed via the debiased sparse partial correlation (DSPC) algorithm using Cytoscape software^11^ with the MetScape plugin^12^. The DSPC algorithm is a statistical algorithm that estimates the partial correlation coefficients between variables, accounting for the sparsity and bias of the data, providing more accurate and reliable results via regularization techniques and debiasing methods. The DSPC algorithm is based on the application of the desparsified graphical lasso model, considering that the number of true connections among the metabolites is significantly smaller than the utilized sample size.

Thus, the DSPC algorithm calculates the strength and relationship between changes in metabolite levels and clinical and anthropometric parameters. We used a previously approved method for the age-adjusted correction of metabolomic data in patient groups that were not balanced according to age^13^.

## Results

### Baseline characteristics of the participants

**Fig. 1:**
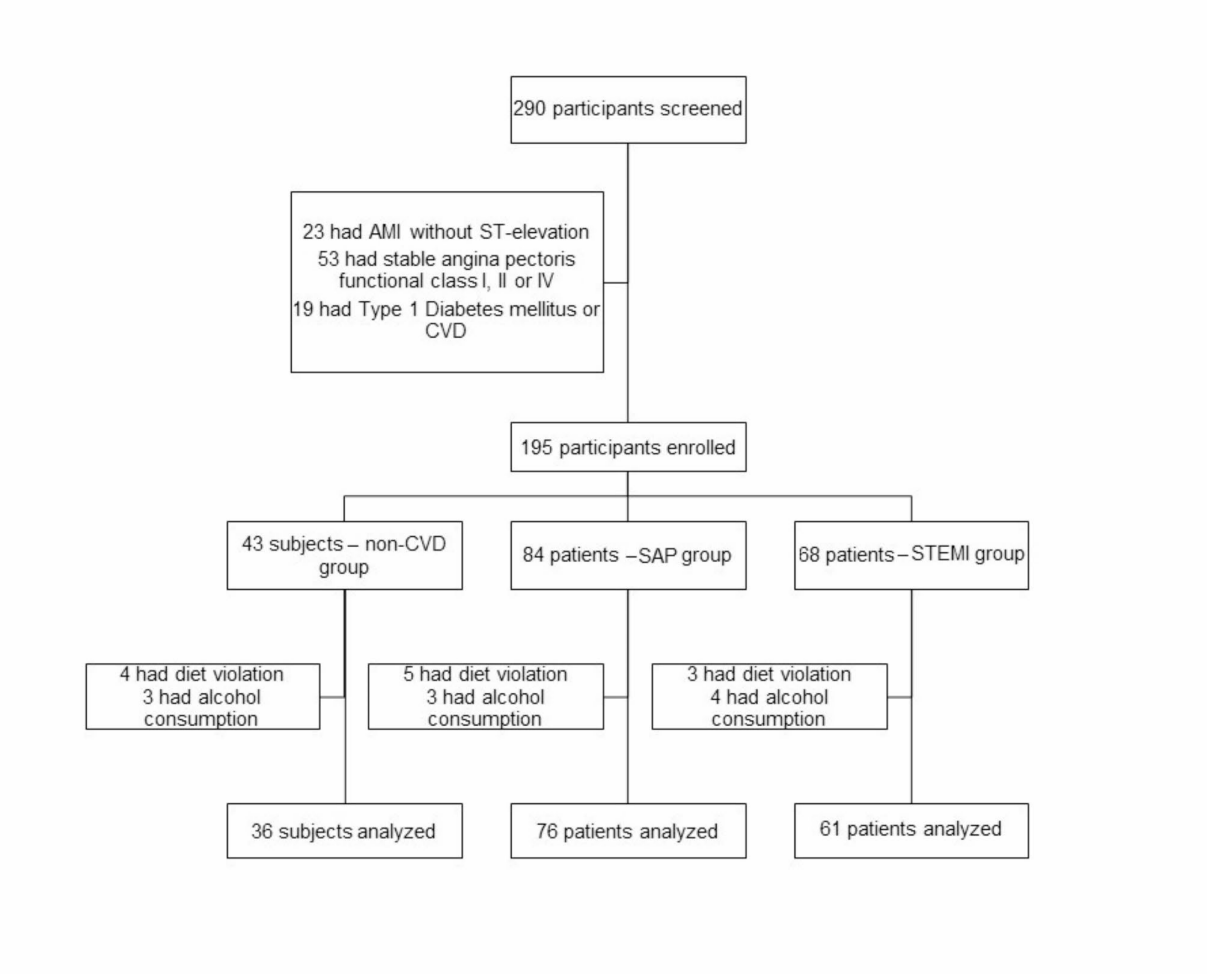
Trial profile.

A total of 290 patients were screened for the study. Ninety-five patients did not meet the inclusion criteria and were ineligible (Fig. 1).

Four patients in the non-CVD group, 5 patients in the SAP group and 3 patients in the STEMI group were excluded from the study because of diet violations (energy drink consumption). Three patients in the non-CVD group, 3 patients in the SAP group and 4 patients in the STEMI group were excluded because of alcohol consumption before blood analysis.

Patients with STEMI had evidence of ST-segment elevation on their qualifying electrocardiogram (at least 2 mm in two contiguous peripheral or precordial leads; the average ST elevation was 3.58 ± 1.96 mm). STEMI patients were treated with intravenous thrombolysis using alteplase or non-immunogenic staphylokinase, followed by percutaneous coronary intervention (PCI) with stenting. Additionally, they received intravenous heparin (70 IE/kg), clopidogrel, acetylsalicylic acid and, if necessary, intravenous fentanyl, morphine or nitro glycerine for chest pain. Patients with SAP were managed with organic nitrates, β-blockers, calcium channel blockers, ACE inhibitors and statins.

Patients diagnosed with SAP had stable angina pectoris functional class III according to the Canadian Cardiovascular Society classification and combined dyslipidaemia characterized by elevated triglyceride levels and decreased HDL-cholesterol levels^14^.

The non-CVD group consisted of adults without any clinical or laboratory signs of cardiovascular pathology or risk factors for SAP.

Information on demographics, medical history, biochemical analysis and patient treatment information was obtained from the hospital database.

Among the included patients, those with STEMI and SAP were older than those in the non-CVD group and had higher BMIs.

Troponin I levels were significantly greater in the STEMI group than in the non-CVD and SAP groups. Lipid analysis revealed that total cholesterol levels were within the normal range in all groups, but high triglyceride levels and low HDL-cholesterol levels, which are characteristic of combined dyslipidaemia, were observed in STEMI and SAP patients.

Increased creatinine levels were noted in the SAP group compared with the non-CVD and STEMI groups; however, the levels were within the normal range. ALT, AST and glucose levels were increased in the STEMI group. Coagulograms revealed that the INR, APTT and fibrinogen levels in all the groups were within the normal ranges.

LVEF was lower in patients with STEMI and SAP than in non-CVD patients.

Detailed information concerning the characteristics of the patients is presented in Table 2.

**Table 2:** Baseline characteristics of the participants.

Variable	Non-CVD group (*n* = 36)	SAP group (*n* = 76)	STEMI group (*n* = 61)	*p* value		
				Non-CVD vs. SAP	Non-CVD vs. STEMI	SAP vs. STEMI
Sex, m/f (%)	29/7 (80/20)	65/11 (86/14)	50/11 (82/18)			
Age, years	34 [26–44]	64 [58–71]	58 [54–66]	< 0.0001	< 0.05	0.10
Height, m	1.67 [1.62–1.72]	1.69 [1.64–1.75]	1.72 [1.68–1.76]	0.21	0.11	0.72
Weight, kg	69.0 [59.0–81.0]	84.4 [70.0–89.0]	85.3 [72.0–90.0]	< 0.0001	< 0.001	0.57
BMI, kg/m^2^	24.6 [20.7–27.4]	29.5 [25.6–31.63]	28.8 [24.9–31.2]	< 0.0001	< 0.01	0.65
Heart rate/min	62.2 ± 10.8	65.8 ± 9.2	75.9 ± 14.7	0.51	< 0.05	< 0.05
Systolic BP, mm Hg	119.5 ± 5.6	120.3 ± 6.9	118.6 ± 8.2	0.82	0.59	0.62
Diastolic BP, mm Hg	75.9 ± 6.2	74.7 ± 5.9	74.7 ± 7.2	0.78	0.65	0.95
STEMI localization						
Anterior wall infarction	n/a	n/a	26 (42.7)	n/a	n/a	n/a
Inferior wall infarction			34 (55.7)			
Other			1 (1.6)			
Troponin I, ng/ml	0.001 [0.00–0.005]	0.05 [0.02–0.07]	0.29 [0.08–1.03]	< 0.05	< 0.001	< 0.05
Total cholesterol, mmol/l	5.27 [4.81–5.93]	5.55 [4.50–6.20]	5.51 [4.62–6.21]	0.48	0.47	0.89
Triglycerides, mmol/l	1.10 [0.76–1.28]	1.73 [0.96–2.18]	1.82 [1.01–2.21]	< 0.01	< 0.01	0.51
LDL cholesterol, mmol/l	3.32 [2.71-4.00]	3.30 [2.92–4.10]	3.52 [3.00-4.22]	0.61	0.74	0.88
VLDL cholesterol, mmol/l	0.51 [0.30–0.69]	1.00 [0.72–1.20]	0.97 [0.61–1.21]	0.01	0.01	0.95
HDL cholesterol, mmol/l	1.51 [1.22–1.78]	1.08 [0.89–1.19]	1.22 [1.01–1.42]	< 0.001	< 0.01	< 0.05
Glucose, mmol/l	4.88 [4.59–5.30]	5.65 [4.70–5.88]	10.1* [7.4–10.9]	< 0.01	< 0.001	< 0.001
Creatinine, µmol/l	89.1 [79.4–97.2]	100.5 [85.5-111.6]	75.5 [62.5–85.6]	0.01	0.01	< 0.001
Urea, mmol/l	6.1 [6.1–6.3]	6.5 [5.3–7.4]	6.1 [5.0–7.1]	0.85	0.66	0.23
ALT, u/l	23.0 [20.0–24.0]	29.0 [20.0–34.5]	51.0 [24.3–60.7]	0.31	< 0.01	< 0.01
AST, u/l	25.0 [23.0–27.0]	36.0 [20.0–36.0]	159.1 [32–232]	0.66	< 0.01	< 0.01
CPK, u/L	10.2 ± 2.8	50.3 ± 12.1	328.4 ± 505.6	< 0.05	< 0.001	< 0.001
CPK-MV, u/L	3.1 ± 1.2	10.8 ± 3.7	39.8 ± 59.9	< 0.05	< 0.001	< 0.001
Potassium, mmol/l	4.63 [4.30–4.90]	4.41 [4.10–4.65]	3.72 [3.35–3.96]	< 0.05	< 0.001	< 0.001
Sodium, mmol/l	142.8 [141–145]	140.0 [138.0-142.2]	142.1 [141.0-143.7]	0.12	0.76	0.08
Calcium, mmol/l	–	2.18 [2.10–2.23]	0.91 [0.81–1.01]	–	–	< 0.01
INR	1.09 [1.05–1.14]	1.23 [1.11–1.28]	1.13 [1.01–1.23]	< 0.05	0.53	0.05
APTT, s	27.2 [25.5–28.6]	31.5 [27.0-34.8]	42.0 [28.9–74.0]	0.19	< 0.05	< 0.05
Fibrinogen, g/L	2.25 [2.05–2.30]	3.28 [2.53–3.74]	3.40 [3.10–4.10]	< 0.05	< 0.01	0.21
LVEF, %	59.8 [60.2–64.5]	53.2 [45.0–60.0]	49.2 [39.0-52.3]	0.08	< 0.01	0.05

Baseline characteristics of the participants, including n (%) or median and interquartile range [Q1; Q3], in the considered groups and corresponding p values indicating statistically significant differences between groups.

*Hyperglycaemia is due to nonfasting blood samples obtained before angiography in STEMI patients.

### Correlations between plasma metabolite levels and biochemical analysis results

Correlation analysis revealed that short-chain acyl carnitines were directly correlated with weight and glucose and AST levels, whereas potassium, calcium, magnesium and prothrombin levels were inversely correlated. Moreover, the levels of medium-chain acylcarnitines were directly correlated with age; creatinine, urea, and calcium levels; and the international normalized ratio (INR) but inversely correlated with LDL cholesterol, glucose, ALT, AST, CPK and prothrombin levels.

Long-chain acylcarnitines were directly correlated with weight, BMI and the INR. Long-chain carnitine levels were inversely correlated with prothrombin, calcium, potassium, glucose, and HDL cholesterol levels. Creatinine is strongly correlated with choline, citrulline, cystathionine, SDMA, 3-aminoisobutyric acid, 3-hydroxyanthranilic acid, 3-hydroxykynurenine, GABA, 5-hydroxytryptophan, acetylcholine, anthranilic acid, aspartic acid, biopterin, 5-hydroxyindoleacetic acid, indole-3-acetic acid, indole-3-carboxaldehyde, indole-3-butyric acid, indole-3-propionic acid, kynurenic acid, kynurenine, melatonin, metanephrine, neopterin, norepinephrine, normetanephrine, quinolinic acid, serotonin, tryptophan, tryptophol, vanillylmandelic acid, xanthurenic acid and Kyn/Trp.

HDL cholesterol was directly correlated with the levels of several amino acids (alanine, arginine, isoleucine, methionine, and threonine), whereas sodium was inversely correlated with leucine, phenylalanine, serine, and valine. In addition, 5HIAA, kynurenine, kynurenic acid, 3-aminoisobutyric acid, and Kyn/Trp were strongly correlated with BMI and urea.

**Fig. 2:**
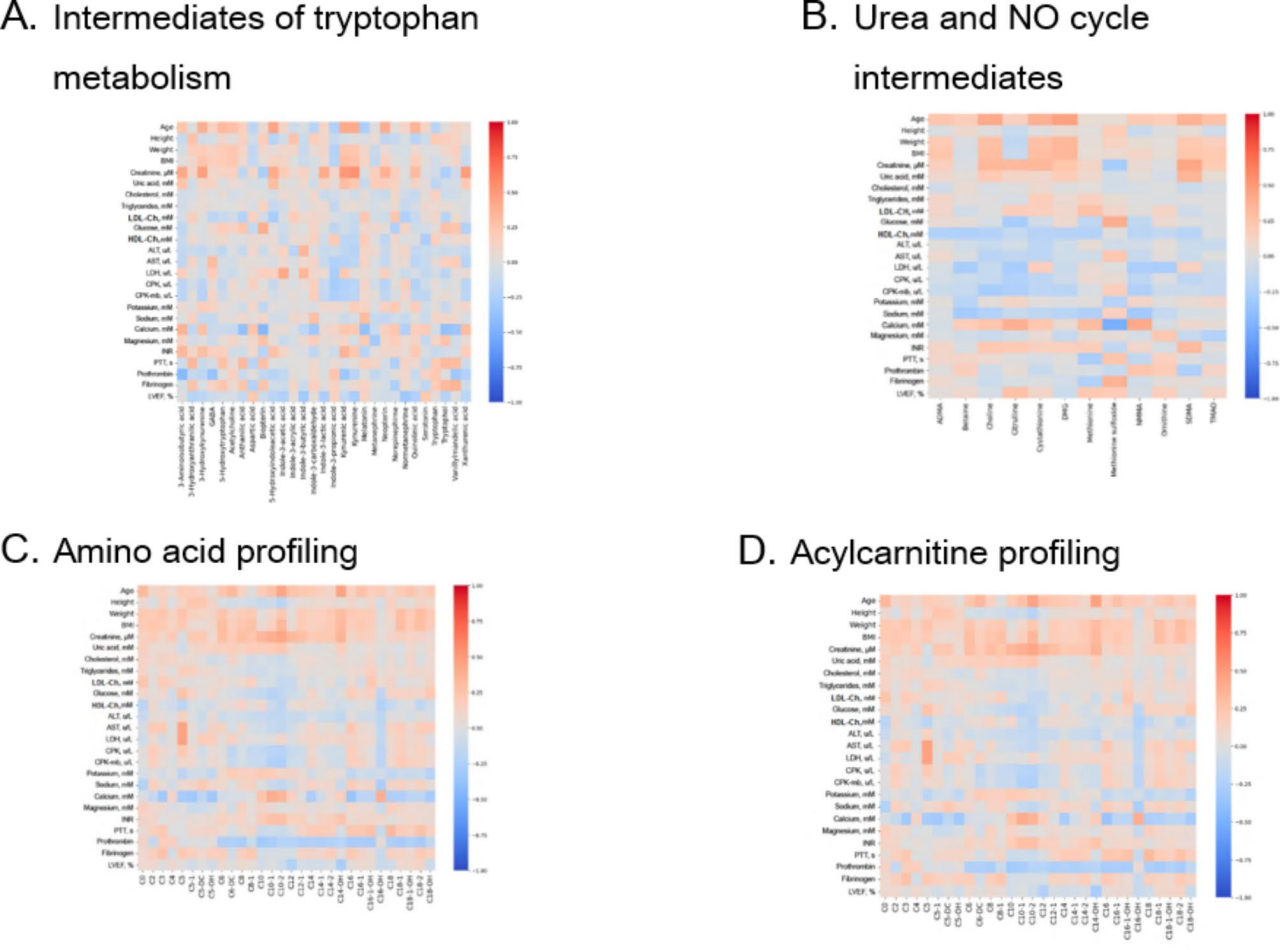
Heatmap correlation matrices of plasma metabolites and the biochemical analysis results.

The correlation heatmaps showing the relationships between the observed concentrations of the metabolites and the corresponding biochemical analysis results are presented in Fig. 2A–D.

### Differences in the metabolic profiles of STEMI patients, SAP patients and non-CVD subjects

For a general overview of the data and outlier exclusion, a principal component analysis (PCA) was performed, and the results revealed that the groups can be partly separated from each other (Supplementary Materials, Figure S1). Using OPLS-DA, the list of metabolites with the highest VIP scores was obtained (Table 3, Supplementary Materials, Table S9, Figure S2).

**Table 3:** List of the metabolites with the highest VIP scores (VIP score > 1.5) obtained from the OPLS-DA.

Metabolite	VIP score
GSG ratio	1.96
Glutamic acid	1.78
Tryptophol	1.70
DMG	1.62
C5	1.62
Vanillylmandelic acid	1.60
Methionine sulfoxide	1.54
C10-2	1.51

**Fig. 3:**
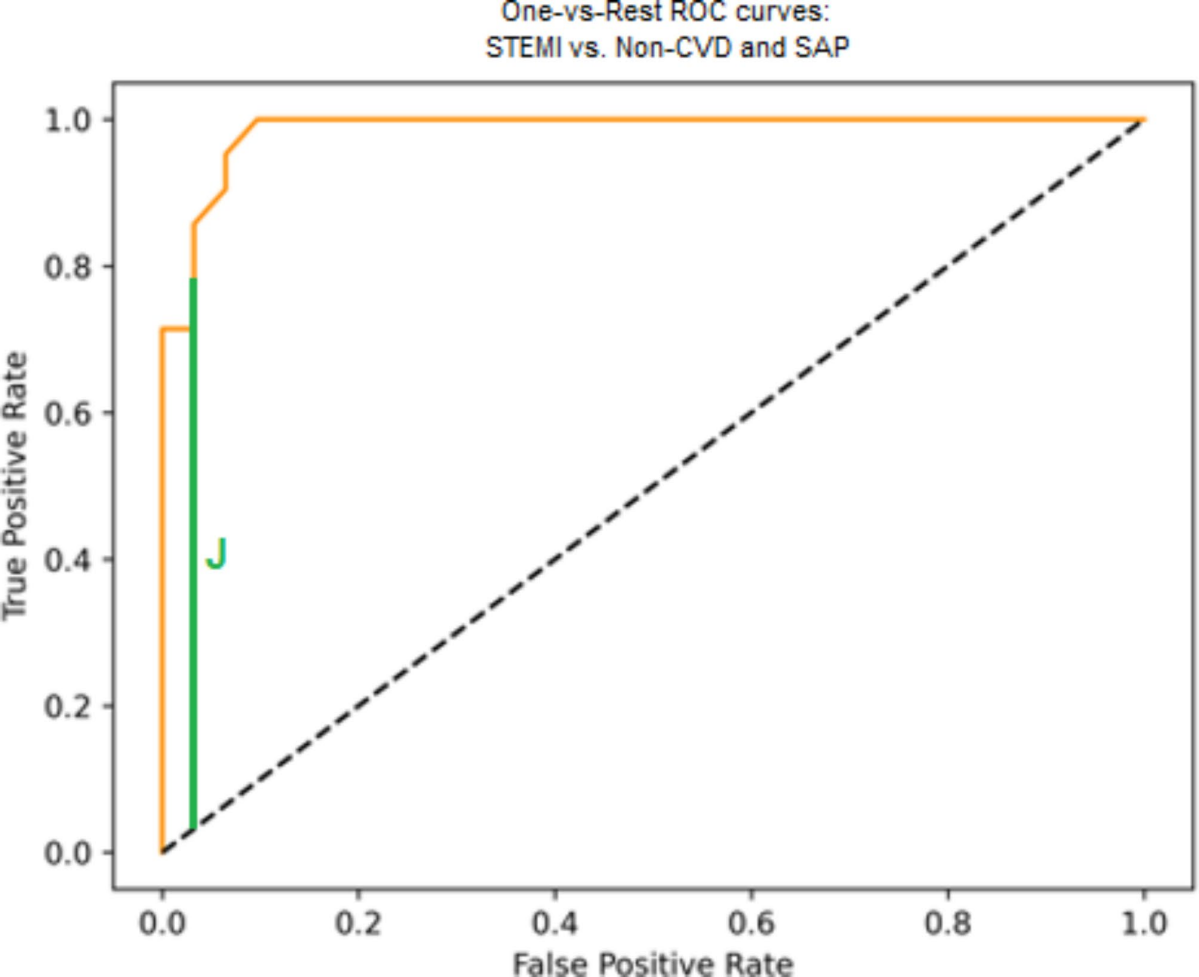
AUC ROC.

Finally, a random forest model was built, and the hyperparameters and quality metrics are presented in Table 4. The AUC ROC curve is presented in Fig. 3.

**Table 4:** Random forest quality metrics.

Hyperparameters of the model	criterion=’entropy’, max_depth = 120, random_state = 42.
Accuracy	0.87
F1 score	0.86
Recall	0.86
Youden index	0.775
AUC ROC	0.95

Confusion matrix	Non-CVD	SAP	STEMI
Non-CVD	19	1	1
SAP	0	9	1
STEMI	2	2	17

The metabolites that were significantly altered among the of patient groups were identified using parametric and nonparametric comparison tests. Table 5 summarizes the information on the significantly altered metabolites, including the metabolite classes, direction of change and adjusted p values.

**Table 5:** Important metabolites selected on the basis of multiple hypothesis comparisons with Bonferroni correction.

Metabolite	Adj. *p* value (among 3 groups)	SAP vs. Non-CVD		STEMI vs. Non-CVD		STEMI vs. SAP	
		Direction (for SAP)	Adj. *p* value	Direction (for STEMI)	Adj. *p* value	Direction (for STEMI)	Adj. *p* value
N-methylmalonamic acid (NMMA)	< 0.0001	Increased	< 0.05	–	–	Decreased	< 0.001
Valine	< 0.01	Increased	< 0.01	–	–	Decreased	< 0.05
Proline	–	–	–	–	–	Increased	< 0.05
Phenylalanine	< 0.00001	Increased	< 0.0001	Increased	< 0.05	–	–
Methionine	< 0.001	–	–	–	–	Decreased	< 0.001
Leucine	< 0.0001	Increased	< 0.01	–	–	Decreased	< 0.05
Isoleucine	< 0.05	Increased	< 0.05	–	–	–	–
Glycine	< 0.01	Decreased	< 0.05	Decreased	< 0.05	Decreased	< 0.05
Asparagine	< 0.01	–	–	–	–	Increased	< 0.01
Aspartic acid	< 0.0001	–	–	Decreased	< 0.001	Decreased	< 0.001
Glutamine	< 0.0001	Increased	< 0.01	Increased	< 0.0001	Increased	< 0.05
Carnitine (C0)	< 0.01	Increased	< 0.01	Increased	< 0.05	–	–
Isovalerylcarnitine (C5)	< 0.0001	Increased	< 0.01	Increased	< 0.0001	Increased	< 0.001
Tiglylcarnitine (C5-1)	–	–	–	Increased	< 0.01	Increased	< 0.05
Adipoylcarnitine (C6-DC)	< 0.01	Increased	< 0.01	–	–	–	–
Decenoylcarnitine (C10-1)	< 0.001	Increased	< 0.05	–	–	Decreased	< 0.0001
Decadienoylcarnitine (C10-2)	< 0.0001	Increased	< 0.001	–	–	Decreased	< 0.0001
Hydroxytetradecanoylcarnitine (C14-OH)	< 0.0001	Increased	< 0.0001	–	–	–	–
Palmitoylcarnitine (C16)	< 0.01	–	–	Increased	< 0.0001	Increased	< 0.01
Hydroxyhexadecanoylсarnitine (C16-OH)	< 0.0001	Increased	< 0.0001	Increased	< 0.01	Decreased	< 0.05
Palmitoleyl carnitine (C16-1)	< 0.05	Increased	< 0.05	Increased	< 0.001	Increased	< 0.001
Oleoylcarnitine (C18-1)		–	–	Increased	< 0.05	–	–
Hydroxystearoylcarnitine (C18-OH)	< 0.0001	Increased	< 0.05	Increased	< 0.0001	Increased	< 0.05
Symmetric dimethylarginine (SDMA)	< 0.01	Increased	< 0.01	Increased	< 0.05	–	–
Methionine sulfoxide	< 0.0001	–	–	Increased	< 0.0001	Increased	< 0.0001
Dimethylglycine (DMG)	< 0.0001	Increased	< 0.0001	Increased	< 0.0001	–	–
Cystathionine	< 0.001	Increased	< 0.001	–	–	Decreased	< 0.01
Citrulline	< 0.01	–	–	–	–	Decreased	< 0.01
Choline	< 0.0001	Increased	< 0.0001		–		–
Vanillylmandelic acid	< 0.001		–	Increased	< 0.01	Increased	< 0.05
Biopterin	< 0.001		–	Increased	< 0.01	Increased	< 0.01
Acetylcholine	< 0.01	Increased	< 0.01	Increased	< 0.05		–
3-Amino isobutyric acid	< 0.01	Increased	< 0.05		–	Decreased	< 0.01
Kynurenine	< 0.01	Increased	< 0.01		–		–
3-Hydroxykynurenine	< 0.001		–		–	Decreased	< 0.001
Kynurenic acid	< 0.0001	Increased	< 0.001		–		–
Anthranilic acid	< 0.0001	Increased	< 0.001	Increased	< 0.0001	Increased	< 0.01
Xanthurenic acid	< 0.01		–		–	Decreased	< 0.01
Tryptophol	< 0.0001		–	Increased	< 0.0001	Increased	< 0.0001
Indole-3-propionic acid	< 0.01		–	Decreased	< 0.01		
Serotonin	< 0.05		–	Increased	< 0.001		–

The data revealed that some metabolites were changed in STEMI patients compared with non-CVD patients. The significantly increased metabolites included amino acids, acylcarnitines, NO-urea cycle metabolites, neurotransmitters, and intermediates of tryptophan metabolism. Regarding amino acids, phenylalanine and glutamine were notably elevated. The increased acylcarnitines included carnitine (C0), isovalerylcarnitine (C5), tiglylcarnitine (C5-1), palmitoylcarnitine (C16), palmitoleyl carnitine (C16-1), hydroxyhexadecanoylсarnitine (C16-OH), oleoylcarnitine (C18-1), and hydroxystearoylcarnitine (C18-OH). Regarding NO-urea cycle metabolites and neurotransmitters, methionine sulfoxide, symmetric dimethylarginine (SDMA), dimethylglycine (DMG), vanillylmandelic acid, acetylcholine, and biopterin were increased. Regarding intermediates of tryptophan metabolism, increased levels of tryptophol, anthranilic acid and serotonin were observed. The significantly decreased metabolites included glycine and aspartic acid (amino acids) and indol-3-propionic acid (tryptophan metabolism).

Significant changes were also found in STEMI patients compared with SAP patients. The significantly increased metabolites included amino acids such as proline, glutamine and asparagine; acylcarnitines such as isovalerylcarnitine (C5), tiglylcarnitine (C5-1), palmitoylcarnitine (C16), palmitoleyl carnitine (C16-1), and hydroxystearoylcarnitine (C18-OH); NO-urea cycle metabolites and neurotransmitters such as methionine sulfoxide, vanillylmandelic acid, and biopterin; and intermediates of tryptophan metabolism such as anthranilic acid and tryptophol. The significantly decreased metabolites included amino acids such as methionine, leucine, valine, N-methylmalonamic acid (NMMA), glycine and aspartic acid; acylcarnitines such as decenoylcarnitine (C10-1), decadienoylcarnitine (C10-2), and hydroxyhexadecanoylсarnitine (C16-OH); NO-urea cycle metabolites and neurotransmitters such as cystathionine, citrulline, and 3-amino isobutyric acid; and intermediates of tryptophan metabolism such as xanthurenic acid and 3-hydroxykynurenine.

The data revealed that some metabolites were also changed in STEMI patients compared with those in SAP patients and non-CVD subjects. The significantly increased metabolites included amino acids such as glutamine; acylcarnitines such as isovalerylcarnitine (C5), tiglylcarnitine (C5-1), palmitoylcarnitine (C16), and palmitoleyl carnitine (C16-1, hydroxystearoylcarnitine (C18-OH)); NO-urea cycle metabolites and neurotransmitters such as biopterin, methionine sulfoxide, and vanillylmandelic acid; and intermediates of tryptophan metabolism such as anthranilic acid and tryptophol. The significantly decreased metabolites included only the amino acids glycine and aspartic acid.

These results indicate that the concentrations of glutamine, isovalerylcarnitine (C5), hydroxystearoylcarnitine (C18-OH) and anthranilic acid are significantly elevated during coronary artery progression (non-CVD → SAP → STEMI), whereas glycine and aspartic acid levels are significantly decreased.

The GSG ratio (glutamine: serine + glycine) also increased with coronary artery progression.

According to OPLS-DA, eight metabolites had VIP scores greater than 1.50 (Table 3). The highest VIP score was associated with a GSG ratio of 1.96.

**Fig. 4:**
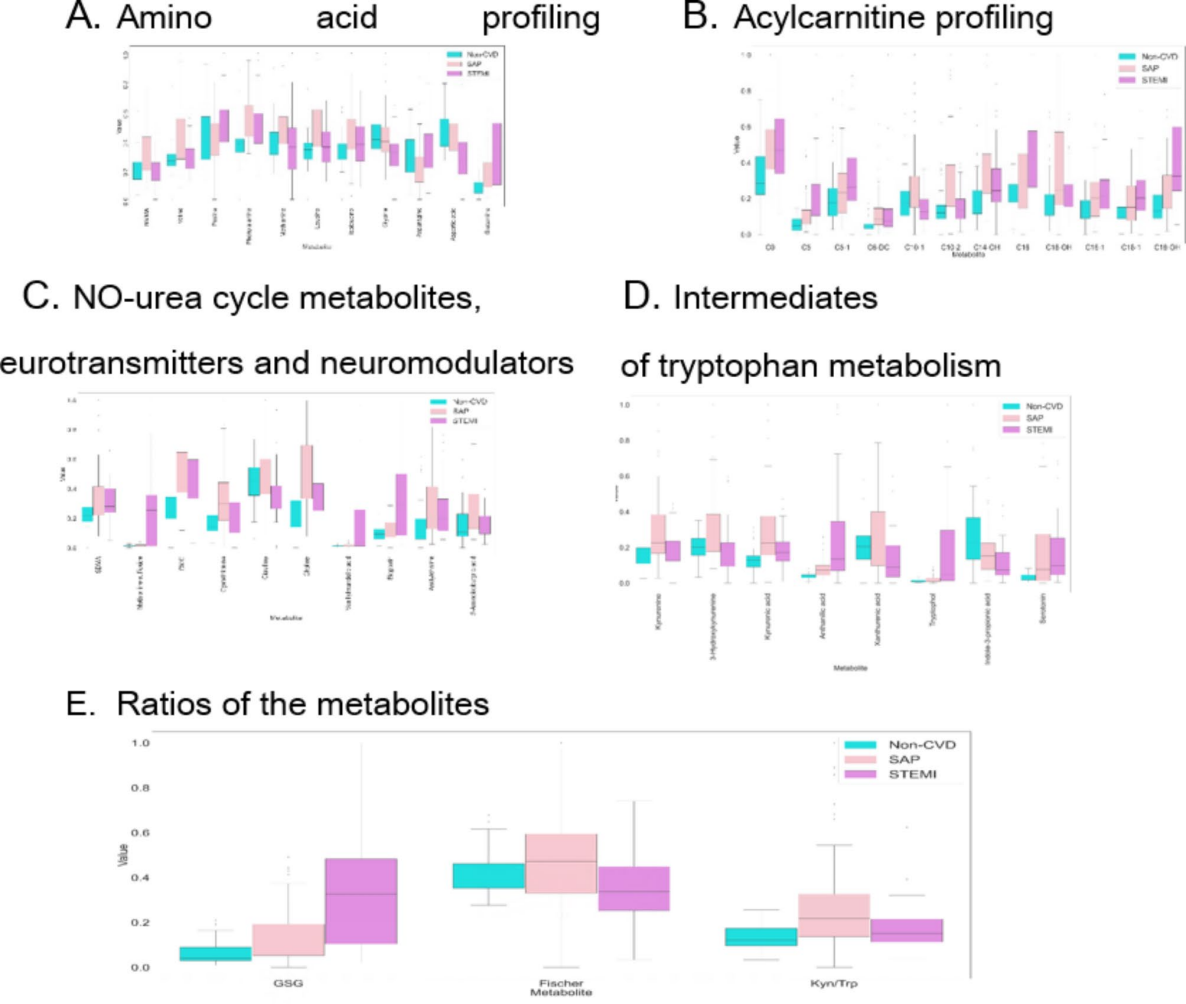
Horizontal bar charts of the significantly altered metabolites among the groups of patients.

A graphical interpretation of the obtained results after min–max normalization is presented in Fig. 4 (A-E) (A – amino acid profiling; B – acylcarnitine profiling; C – metabolites of the NO-urea cycle, neurotransmitters and neuromodulators; D – tryptophan metabolism intermediates; E – ratios of the metabolites).

**Fig. 5:**
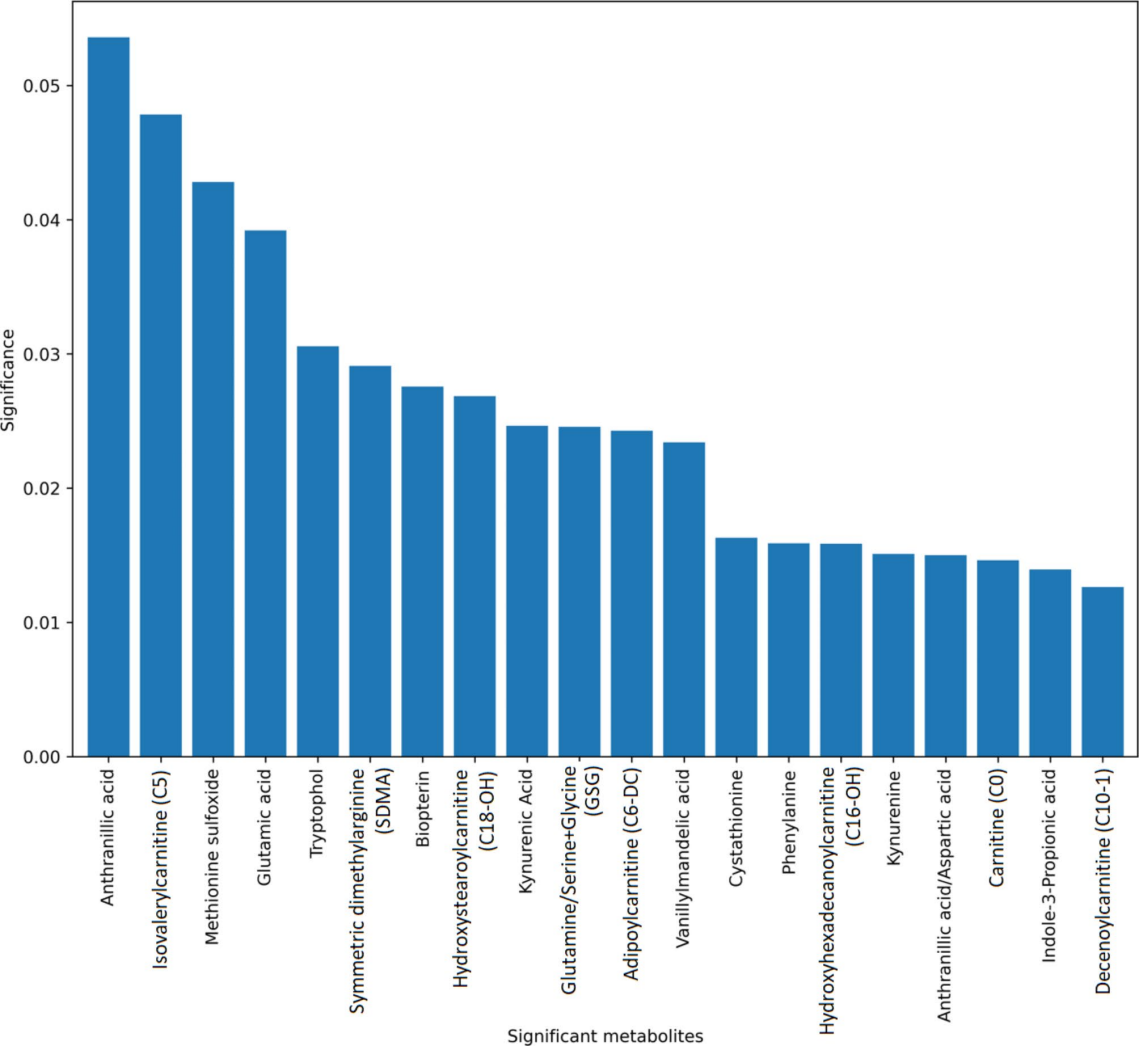
Histogram representing metabolites with the greatest impact on the STEMI diagnostics established using the random forest model.

The most significant metabolites in the STEMI patients based on the RF model are presented in Fig. 5. The compounds with the greatest impact on STEMI diagnosis included anthranilic acid, isovalerylcarnitine (C5) and methionine sulfoxide.

**Fig. 6:**
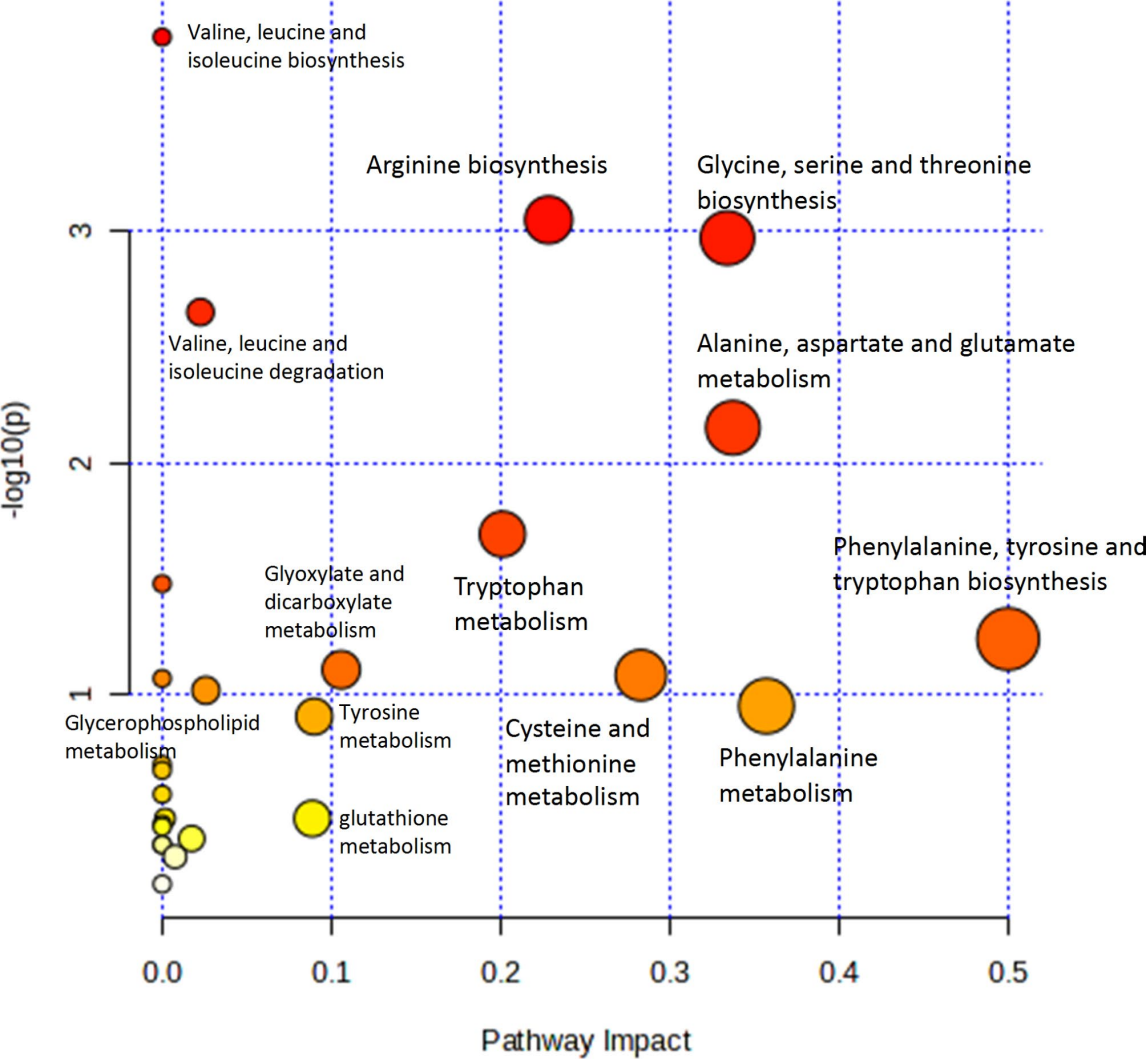
Pathway analysis results. The pathways are shown in accordance with p values (y-axis) obtained from the pathway enrichment analysis, whereas pathway impact values (x-axis) are responsible for topology analysis. The colours of the pathways reflect the p value. Red corresponds to low statistical significance, and yellow indicates the highest statistical significance.

The biochemical connections between significantly different pathways based on the results of the metabolomic profiling are presented in Fig. 6. The pathways are presented with p values (y-axis) obtained from the pathway enrichment analysis, whereas pathway impact values (x-axis) were obtained from topology analysis. The most impactful pathways are the phenylalanine, tyrosine, and tryptophan biosynthesis pathways; the glycine, serine, and threonine biosynthesis pathways; tryptophan metabolism; arginine biosynthesis; and alanine, aspartate and glutamate metabolism.

### Weighted correlation network analysis

**Fig. 7:**
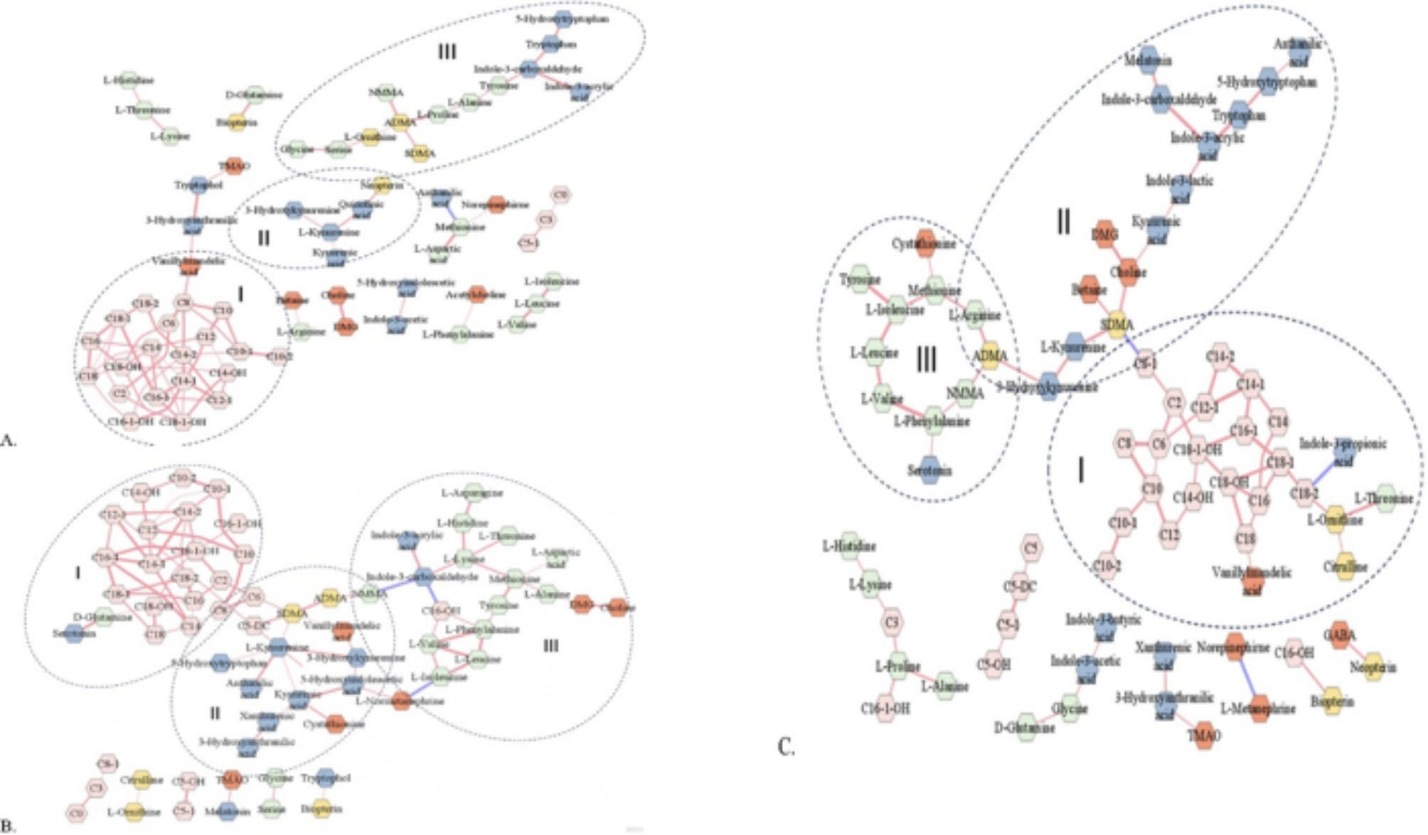
Weighted correlation networks: **A** – STEMI group; **B** – SAP group; **C** – non-CVD group. I, II, III – metabolite clusters.

Weighted correlation network analysis was performed to identify relationships between the profiled metabolites and quantified clinical factors. These findings explained the functional metabolic modules of the measured metabolites in the plasma of STEMI, SAP and non-CVD patients (Fig. 7A, B and C, respectively) and their correlation with anthropometric and clinical factors.

Three specific modules (I, II, III) were found, and it was possible to identify the main metabolite hubs in each module.

The first module (Fig. 7I) of medium- and long-chain acylcarnitines was similar among all three groups of patients. The number of edges of this module in STEMI patients was significantly lower than that in SAP patients and non-CVD subjects, which may be explained by the alternative activity of these metabolites in STEMI patients.

The second module consisted of the kynurenine metabolic pathway (Fig. 7II). In STEMI patients, tryptophan pathway metabolites are classified into two categories: one is associated with amino acids, and the second is associated with neopterin. The key nodes included indole-3-carboxaldehyde, kynurenine and indole-3-acrylic acid in the STEMI, SAP and non-CVD groups, respectively.

The third module consisted of amino acids (Fig. 7III). Among these metabolites, the direct correlation between BCAAs was relatively the same in STEMI patients, SAP patients and non-CVD patients.

Additional information regarding the characteristics of the networks is presented in the Supplementary Material (Table S10).

On the basis of the differences in the constructed networks, new significant ratios of the metabolites were found. Among the 22 established significantly altered metabolite ratios, 13 ratios between STEMI patients and non-CVD patients and 17 ratios between STEMI patients and SAP patients were found. Seven significant ratios were consistent between STEMI and non-CVD patients and STEMI and SAP patients, including anthranilic acid: kynurenine; anthranilic acid: methionine; anthranilic acid: tryptophol; biopterin: methionine sulfoxide; biopterin: metanephrine; aspartic acid: biopterin; and hydroxystearoylcarnitine (C18-OH): indole-3-propionic acid. Two ratios were consistent among the STEMI, SAP and non-CVD groups, namely, anthranilic acid: aspartic acid and GSG (glutamine: serine + glycine) (Table 6). These ratios may function as new potential biomarkers for STEMI patients.

**Table 6:** Significantly altered metabolite ratios based on the conducted weighted correlation network analysis.

Ratio	*p* value non-CVD – SAP	*p* value STEMI – non-CVD	*p* value STEMI – SAP	*p* value Kruskal
Anthranilic acid: Aspartic acid	< 0.001	< 0.00001	< 0.001	< 0.00001
Anthranilic acid: Kynurenine		< 0.00001	< 0.00001	< 0.00001
Anthranilic acid: Methionine		< 0.001	< 0.001	< 0.0001
3OH Anthranilic acid: Tryptophol		< 0.00001	< 0.00001	< 0.00001
Biopterin: Methionine sulfoxide		< 0.00001	< 0.00001	< 0.00001
Biopterin: Metanephrine		< 0.01	< 0.01	< 0.01
Aspartic acid: Biopterin		< 0.001	< 0.001	< 0.0001
GSG (Glu: Ser + Gly)	< 0.01	< 0.00001	< 0.0001	< 0.00001
Hydroxystearoylcarnitine (C18-OH): Indole-3-propionic acid		< 0.0001	< 0.01	< 0.0001
Tryptophan:5OH Tryptophan	< 0.0001	< 0.0001		< 0.00001
Citrulline: Ornithine		< 0.01		< 0.01
Lauroylcarnitine (C12):Hydroxytetra-decanoylcarnitine (C14-OH)	< 0.001	< 0.01		< 0.01
ADMA: NNMA			< 0.0001	< 0.001
ADMA: Proline			< 0.05	< 0.05
Arginine: ADMA		< 0.001		< 0.01
Kynurenine: Tryptophan	< 0.01		< 0.05	< 0.001
NMMA: Indole-3-carboxaldehyde	< 0.05		< 0.001	< 0.001
3OH Kynurenine: Kynurenine			< 0.01	< 0.01
Norepinephrine: Metanephrine			< 0.01	< 0.01
Asparagine: Histidine			< 0.01	< 0.05
Fischer (Leu + Ile + Val): (Tyr + Phe)			< 0.01	< 0.05
Aspartic acid Methionine	< 0.01			< 0.05
*n* = 22	*n* = 7	*n* = 13	*n* = 17	

**Fig. 8:**
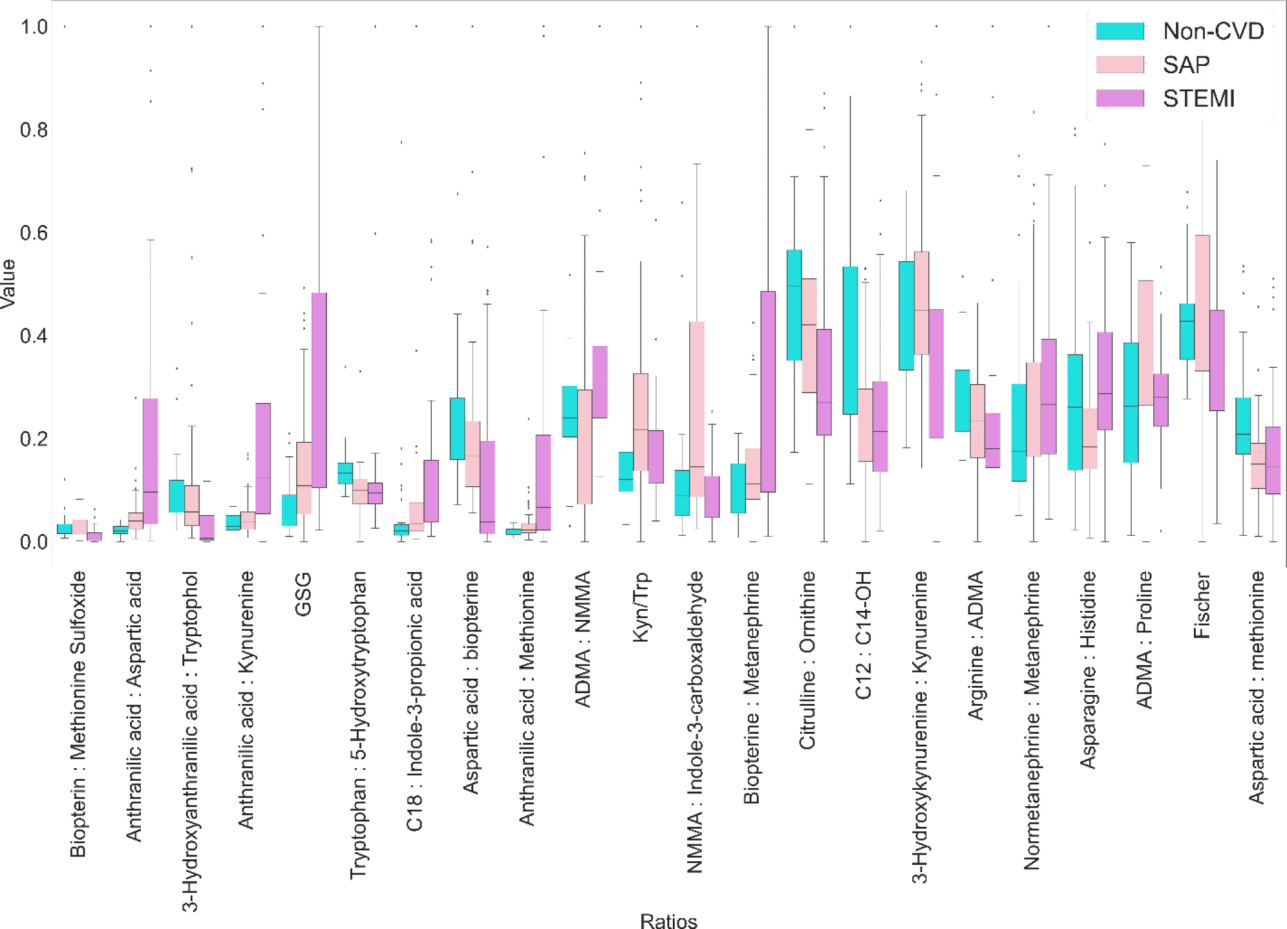
Horizontal bar charts of the significantly altered ratios of the metabolites based on the weighted correlation network analysis results.

A graphical interpretation of the most significant ratios is presented in Fig. 8.

## Discussion

The present study is the first to perform targeted metabolomic profiling in patients with STEMI. We identified 36 significantly changed metabolites in STEMI patients. Increased levels of four amino acids, eight acylcarnitines, six metabolites of the NO–urea cycle and neurotransmitters and three intermediates of tryptophan metabolism were observed. The following metabolites exhibited decreased levels: six amino acids, three acylcarnitines, three metabolites of the NO–urea cycle and neurotransmitters and three intermediates of tryptophan metabolism.

Previous studies have linked changes in metabolite concentrations with MI development. In a meta-analysis of six cohort metabolomic studies, 7897 individuals whose blood metabolic profiles included 1428 metabolites were explored, and 1373 MI cases were identified during 9.4 years of follow-up. Fifty-six metabolites, including 21 lipids and 17 amino acids, were associated with MI after adjusting for multiple testing. The greatest increase in risk was observed for the carbohydrate mannitol/sorbitol, whereas the greatest decrease in risk was noted for glutamine^15^.

Regarding study limitations, the authors note that the MI clinical definition and its types varied in each cohort depending on the protocol for data collection. Metabolomic profiling was conducted using different metabolomic platforms, metabolite sampling and detection times were not unified, and concomitant therapy was not considered. For the first time, a predictive platform was used to identify the importance of a balanced amino acid profile in an organism as well as the protective role of nonessential amino acids and the multiple roles of lipids in the risk of MI.

In our study, glutamine levels were significantly increased in STEMI patients, and we hypothesize that these changes may have been caused by acute myocardial ischaemia.

Few studies have investigated STEMI among patients with MI, acute coronary syndrome and myocardial ischaemia. In a study of 23 young STEMI patients (under 45 years old) compared with elderly STEMI patients (65–80 years old), four principal pathways exhibited significant differences in metabolites according to untargeted metabolomics profiling. Among the sphingolipid, glycerophospholipid, glycine/serine/threonine, and arginine/proline pathways, the sphingolipid metabolomic pathway was the most altered. Sphingolipid metabolism also had the highest predictive value for major adverse cardiovascular events in young STEMI patients at the one-year follow-up^16^.

In a study of 30 STEMI patients using untargeted metabolomics profiling, 19 metabolites were identified that distinguished STEMI patients from unstable angina patients and healthy subjects. Among these metabolites, hydrogen sulphide (H_2_S) is an endogenous gasotransmitter with cardioprotective potential. A highly sensitive immunoassay (ELISA) confirmed the significant increase in H_2_S in STEMI patients compared with unstable angina patients and healthy controls^17^.

The main difference between our study and the abovementioned trial was elevated levels of the amino acids glycine, valine and leucine. However, in our study, the levels of these metabolites significantly decreased, especially glycine. Acylcarnitine profiling revealed the same result as increased levels of carnitine (C0) were detected.

In 22 patients with left main coronary artery disease (LMCAD) with STEMI versus 22 non-LMCAD STEMI patients and healthy controls, 9-cis-retinoic acid (9cRA), the main metabolite of retinol metabolism, was the strongest discriminator of LMCAD patients based on the untargeted metabolomics profiling results. The pathogenesis of LMCAD is not well understood. The identified biomarker 9cRA provides insight into the diagnosis of this disease in the clinic but is not the parameter with the greatest ability to discriminate STEMI patients^18^. In this study, the phospholipid metabolic pathway was also highly involved in the pathogenesis of STEMI patients with and without LMCAD.

We found that the significant changes in tryptophan metabolism in STEMI patients—the increase in anthranilic acid and tryptophol and decrease in xanthurenic acid and 3-OH-kynurenine—may play important roles in STEMI pathogenesis.

The abovementioned metabolites are related to the kynurenine metabolic pathway (KMP), representing the main route of tryptophan catabolism. Significant changes in the concentrations of key KMP intermediates (kynurenine and kynurenic acid) may be related to the activation of the enzymes indoleamine 2,3-dioxygenase (IDO) and tryptophan 2,3-dioxygenase (TDO)^19,20^. We hypothesized that the decreased levels of the end products of 3-OH kynurenine and xanthurenic acid may be explained by the decreased activity of these enzymes.

The endogenous neurotransmitter norepinephrine was significantly decreased in STEMI patients, whereas its end-product, vanillylmandelic acid (VMA), was significantly increased. A significant decrease in plasma norepinephrine concentrations may be associated with an increase in its cellular uptake in STEMI patients.

We identified significant alterations in the concentrations of acetylcholine in STEMI patients. The level of choline, the acetylcholine precursor, was significantly elevated exclusively in SAP patients, whereas in STEMI patients, its level was equal to that in non-CVD subjects. Elevated choline levels serve as a marker of unfavourable cardiovascular risk profiles and MI^21^.

In the present study, we revealed impaired regulation of the homocysteine-methionine cycle, including significantly decreased levels of methionine in STEMI patients and increased levels of cystathionine in SAP patients. Methionine sulfoxide was significantly elevated in STEMI patients possibly because of methionine oxidation. Elevated cystathionine levels in SAP patients are linked to oxidative damage and impaired endothelial function^22^.

Dimethylglycine (DMG), obtained from betaine upon the remethylation of homocysteine to methionine, was significantly elevated in STEMI and SAP patients^23^. Numerous studies have identified DMG as a major marker of myocardial infarction in patients with suspected or established coronary heart disease^24^. According to our data, increased DMG levels may be related to the significantly decreased glycine levels.

The present study revealed that short-chain and long-chain acylcarnitines were significantly increased in STEMI patients. Acylcarnitines play a significant role in myocardial metabolism and are mainly responsible for the mitochondrial β-oxidation of long‐chain fatty acids and, therefore, for energy production. Numerous studies have linked disturbances in acylcarnitines to various cardiovascular disorders and type 2 diabetes mellitus^7,25–27^.

In STEMI patients, aspartic acid levels are significantly decreased, which may contribute to dysregulation of the malate/aspartate shuttle. This pathway is responsible for the recycling of glycolysis-produced NADH through the mitochondria into cytosolic NAD+, maintaining glycolytic activity. Aspartate amino transferase (AST) is responsible for the transformation of aspartate to oxaloacetate, explaining the reduction in aspartate levels^28,29^. Therefore, aspartate metabolism may influence the Krebs cycle during acute ischaemia in the context of STEMI.

**Fig. 9:**
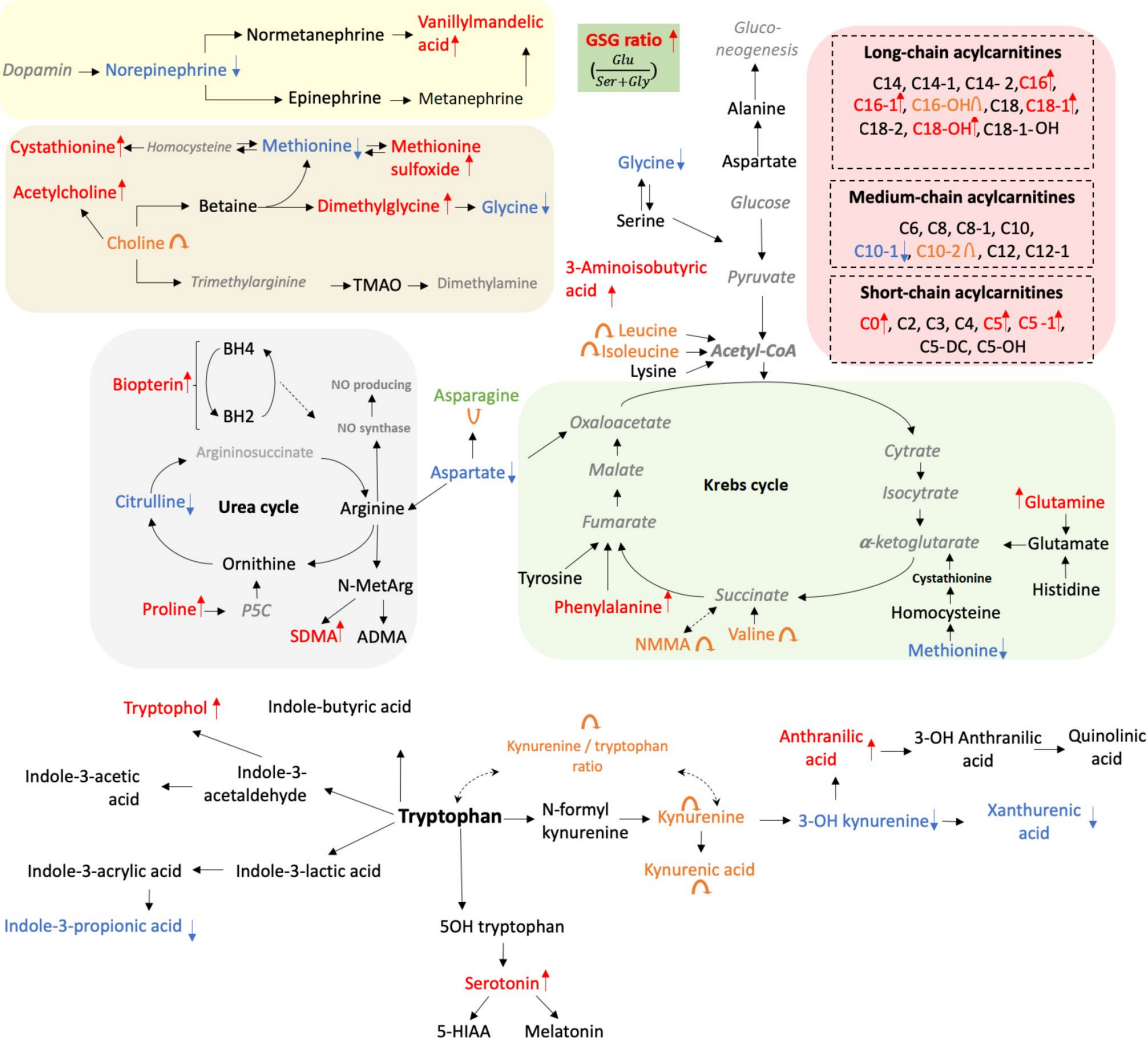
Significantly altered metabolites and metabolic pathways in STEMI pathogenesis.

Figure 9 summarizes the metabolic pathways affected by the identified significantly altered metabolites.

The above pathway analysis and interpretation of the metabolomic profiles were based on the mapping of the identified significantly altered metabolites to preliminarily defined pathways obtained from metabolic databases, such as KEGG^30^ or MetaCyc^31^. Weighted correlation network analysis is an alternative powerful tool for the identification of systemic metabolic changes that are often undetectable when only changes in metabolite levels in database pathway information are used^32^. In the present study, weighted correlation network analysis was performed using the DSPC algorithm, and a normalized approach was created for handling high-dimensional MS-based metabolomic profiling data^33^. Two ratios (anthranilic acid: aspartic acid and GSG) were consistent among the three groups: STEMI, SAP and non-CVD. With respect to the anthranilic acid: aspartic acid ratio, we detected increased anthranilic acid and decreased aspartic acid levels. We hypothesize that the tryptophan metabolic pathway may be connected with metabolites of the Krebs and NO cycles. Anthranilic acid was directly correlated with methionine and aspartic acid. With respect to the GSG ratio, we detected increased levels of glutamine and decreased serine + glycine levels. These ratios were significantly increased in the STEMI group.

The main advantage of this study is the use of targeted metabolomic profiling, which provides new insights into STEMI pathogenesis. The application of machine learning, sophisticated statistical methods and weighted correlation network analysis minimized the bias of the measurements. The multiple measurements in each subject made the statistical comparison much stronger^34^. New significant ratios of the metabolites were identified, and these ratios may serve as new potential biomarkers of STEMI.

This study is not without limitations. This study included a relatively small number of patients with STEMI. Metabolomics profiling of STEMI patients was performed in this study; however, metabolomics profiling in patients with non-STEMI and unstable angina is also necessary. In future studies, it will also be necessary to distinguish STEMI patients with anterior versus posterior wall myocardial infarction given that differences in the metabolomic profile between ST-segment elevation myocardial infarction (STEMI) patients with different localizations.

In this trial, type 1 diabetes was an exclusion criterion, and there were no patients with type 2 diabetes. The number of identified metabolites may not be specific for MI because they may overlap with diabetes, obesity and metabolic syndrome. MI may have similar risk factors as diabetes does. Taken together, further studies in patients with MI and type 2 diabetes are necessary.

Previously identified metabolomic changes in STEMI patients, such as changes in 9-cis-retinoic acid^18^, sphingolipids^16^, hydrogen sulphide^17^ and phospholipids, should be investigated in our future trials.

## Conclusions

In conclusion, our comprehensive study highlights the intricate metabolic changes associated with STEMI and SAP, offering valuable insights into the underlying metabolic dysregulation. Using targeted metabolomic profiling of DSPC and statistical analyses, we identified significant differences in metabolite levels among STEMI patients, SAP patients and non-CVD patients. These changes encompassed various metabolic pathways, including amino acid, acylcarnitine, NO-urea cycle, neurotransmitter and tryptophan catabolism pathways.

Our study went beyond traditional metabolomic analyses by employing weighted correlation network analysis. This network-based approach revealed subtle but crucial alterations in metabolic modules among patient groups, enhancing our understanding of systemic metabolomic changes in patients with STEMI.

In summary, our research contributes to the growing body of knowledge on STEMI pathogenesis and emphasizes the importance of metabolomic profiling, machine learning and network-based analysis.

## Electronic supplementary material

Below is the link to the electronic supplementary material.

Supplementary Material 1

## Data Availability

All the data generated and analysed during this study are included in this published article and its supplementary materials and are available at www.ebi.ac.uk/metabolights/MTBLS10886.

## Declarations

Correspondence and requests for materials should be addressed to S.S.M.
