# Supplementary material for: Targeted metabolomic profiling of acute ST-segment elevation myocardial infarction
Source: Sci Rep. 2024 Oct 11;14:23838. doi: 10.1038/s41598-024-75635-3 (PMC11470145; doi:10.1038/s41598-024-75635-3)

**Supplementary material for the article**

**Targeted metabolomic profiling of acute ST-segment elevation myocardial infarction**

Table S1. Method for tryptophan metabolism intermediates

| Chromatographic separation | |
| --- | --- |
| Equipment | HPLC-MS/MS Agilent 1200 6450 C |
| Column | Discovery PFP HS F5 2.1 × 150, 3µm |
| Column temperature | 40 ^0^С |
| Mobile phases | А: 0.1% HCOOH in water  В: 0.1% HCOOH in acetonitrile |
| Flow rate | 0.4 ml/min |
| Gradient elution | 0.0 min – 1% B 10.0 min – 90% B  4.0 min – 20% B 10.1 min – 1% B  9.0 min – 90% B 12.0 min – 1% B |
| Mass spectrometric determination | |
| Ionization | HESI (Agilent Jet Stream) |
| Gas Temperature | 300 ºС |
| Gas Flow | 8 L/min |
| Nebulizer | 20 psi |
| Sheath Gas Temp | 300 ºС |
| Sheath Gas Flow | 10 L/min |
| Capillary Voltage (Positive) | 3500 V |
| Capillary Voltage (Positive) | 2500 V |
| Mode of Analysis | Dynamic MRM, Cycle time 500ms |

Table S2. Parameters for amino acid profiling

| Chromatographic separation | |
| --- | --- |
| Equipment | HPLC-MS/MS Agilent 1200 6450 C |
| Column | Waters ACQUITY BEH C18 column 1.7 μm, 100 mm × 2.1 mm (Waters, USA) |
| Column temperature | 40 ^0^С |
| Mobile phases | А: 0.1% HCOOH in water  В: 0.1% HCOOH in acetonitrile |
| Flow rate | 0.3 ml/min |
| Gradient elution | 0.0 min – 1% B 8.0 min – 90% B  3.0 min – 20% B 8.1 min – 1% B  5.0 min – 90% B 12.0 min – 1% B |

| Mass spectrometric determination | |
| --- | --- |
| Ionization | HESI+ (Agilent Jet Stream) |
| Source Temperature | 150 ºС |
| Dwell time | 0.019–0.025 sec |
| collision gas medium | nitrogen |
| Capillary Voltage | 2 KV |
| Mode of Analysis | Dynamic MRM |

Table S3. Parameters for acylcarnitine profiling

| Chromatographic separation | |
| --- | --- |
| Equipment | HPLC-MS/MS Agilent 1200 6450 C |
| Column | Waters ACQUITY BEH C18 column 1.7 μm, 100 mm × 2.1 mm (Waters, USA) |
| Column temperature | 40 ^0^С |
| Mobile phases | А: 0.1% HCOOH in water  В: 0.1% HCOOH in acetonitrile |
| Flow rate | 0.3 ml/min |
| Gradient elution | 0.0 min – 1% B 8.0 min – 90% B  3.0 min – 20% B 8.1 min – 1% B  5.0 min – 90% B 12.0 min – 1% B |
| Mass-spectrometric determination | |

| Ionization | HESI+ (Agilent Jet Stream) |
| --- | --- |
| Source Temperature | 150 ºС |
| desolvation temperature | 10 L/min |
| Source gas flow rate | 10 L/hr |
| Desolvation gas flow rate | 1000 L/hr |
| Dwell time | 20 ms |
| collision gas medium | nitrogen |
| Capillary Voltage | 1 KV |
| Cone voltage | 19V |
| Mode of Analysis | Dynamic MRM |

Table S4. Parameters for ADMA and SDMA profiling

| Chromatographic separation | |
| --- | --- |
| Equipment | HPLC-MS/MS Agilent 1200 6450 C |
| Column | Acquity BEH C18 2.1 × 50, 1.7 µm |
| Column temperature | 40 ^0^С |
| Mobile phases | А: 2 mM ammonia formiate 0.015% heptafluorobutanoic acid in water  В: 2 mM ammonia formiate 0.015% heptafluorobutanoic acid in methanol |
| Flow rate | 0.3 ml/min |
| Gradient elution | 1.0 min – 1% B 11.0 min – 90% B  5.0 min – 20% B 11.1 min – 1% B  6.0 min – 90% B 15.0 min – 1% B |

Table S5. MRM transitions for tryptophan metabolism intermediates

| Compound | Retention time | Registered ions | | Fragmentor | Dissociation energy |
| --- | --- | --- | --- | --- | --- |
|  |  | Parent | daughter |  |  |
| 1-Methyltryptophan | 4.2 | 219 | 146 | 90 | 20 |
| 2-hydroxynicotinic acid | 4.1 | 140 | 122 | 100 | 15 |
| 3-Aminoisobutyric acid | 2.3 | 104 | 57 | 90 | 15 |
| 3-Hydroxyanthranilic acid | 5.1 | 154 | 136 | 90 | 10 |
| 3-Hydroxykynurenine | 3.9 | 225 | 162; 110 | 90 | 22; 17 |
| 3-Hydroxyanthranilic-d3 acid | 5.0 | 157 | 139.1 | 90 | 10 |
| 3-Hydroxykynurenine 13C2, 15N | 3.9 | 228.1 | 110.1 | 90 | 17 |
| 4-Aminobutyric acid | 2.0 | 104 | 87; 69 | 90 | 10; 15 |
| 4-Aminobutyric-d6 acid | 2.0 | 110.1 | 93.2; 73.2 | 90 | 10; 15 |
| 5-Hydroxytryptophan | 5.4 | 221 | 204; 162 | 90 | 10; 20 |
| 5-Hydroxytryptophol | 5.9 | 178 | 160 | 100 | 15 |
| 5-Methoxytryptamine | 7.1 | 191 | 174 | 90 | 10 |
| 6-Hydroxymelatonin | 6.5 | 249 | 190 | 110 | 15 |
| Acetylcholine | 5.2 | 146 | 87; 60; 43 | 90 | 15; 10; 30 |
| Anthranilic acid | 6.6 | 138.1 | 120.1; 92 | 95 | 10; 20 |
| Asparagine | 1.1 | 133 | 87; 74 | 100 | 20 |
| Aspartic acid | 1.1 | 134 | 87; 74 | 100 | 10; 15 |
| Biopterin | 2.4 | 238.1 | 194; 178 | 90 | 20 |
| Biopterin-d3 | 2.4 | 241 | 194 | 90 | 20 |
| Choline | 2.2 | 104 | 60 | 90 | 20 |
| Citrulline | 1.3 | 176 | 113; 70 | 90 | 20; 30 |
| Cortisol | 7.3 | 363 | 327; 121 | 120 | 10; 20 |
| Dopamine | 4.8 | 154 | 119; 91 | 90 | 10 |
| Dopamine-d4 | 4.9 | 158.1 | 123.1; 95.1 | 90 | 10; 30 |
| Epinephrine | 3.6 | 184 | 166; 151 | 90 | 5; 25 |
| Glutamic acid | 1.3 | 148 | 84; 56 | 100 | 15; 30 |
| Glutamine | 1.2 | 147 | 130; 84 | 100 | 10; 15 |
| HIAA | 6.1 | 192.1 | 146.1 | 100 | 20 |
| HIAA-d5 | 6.2 | 197 | 150.1 | 100 | 20 |
| Homovanillic acid | 6.3 | 181.1 | 137; 122 | 70 | 4; 16 |
| Indole-3-acetic acid | 7.2 | 176.1 | 130.1 | 95 | 20 |
| Indole-3-acetic-d4 acid | 7.2 | 180.1 | 133.1 | 95 | 20 |
| Indole-3-acryllic acid | 7.3 | 188.1 | 170; 115 | 100 | 20; 25 |
| Indole-3-butyric acid | 7.7 | 204.2 | 144.2; 130.1 | 100 | 25 |
| Indole-3-butyric-d4 acid | 7.8 | 208 | 132 | 100 | 25 |
| Indole-3-carboxaldehyde | 7.2 | 146.1 | 118.1; 91.1 | 95 | 15; 30 |
| Indole-3-carboxaldehyde-13C8 | 7.3 | 154.1 | 126.1; 98.1 | 95 | 30 |
| Indole-3-lactic acid | 6.8 | 206.1 | 118.1 | 95 | 22 |
| Indole-3-lactic-d5 acid | 6.9 | 211 | 122 | 95 | 22 |
| Indole-3-propionic acid | 7.5 | 190.1 | 130.1 | 95 | 20 |
| Indole-3-propionic-d2 acid | 7.6 | 192.1 | 130.1 | 95 | 20 |
| Kynurenic acid | 7.1 | 190.1 | 144.1 | 95 | 20 |
| Kynurenic-d5 acid | 6.7 | 195.1 | 149.1 | 95 | 30 |
| Kynurenine | 5.9 | 209.1 | 146; 94 | 95 | 30; 10 |
| Kynurenine-d4 | 5.9 | 213 | 98.1 | 95 | 10 |
| L-DOPA | 3.0 | 198 | 181; 152 | 100 | 10 |
| L-Tryptophan | 7.3 | 203 | 142; 116 | 100 | 15 |
| L-Tryptophan-d5 | 6.7 | 208.3 | 164.3; 120.1 | 100 | 15 |
| Melatonin | 7.2 | 233 | 174 | 100 | 15 |
| Melatonin-d4 | 7.2 | 237 | 178 | 120 | 10 |
| Metanephrine | 6.0 | 198 | 165; 148 | 90 | 20; 25 |
| N-acetyl-5-hydroxytryptamine | 6.2 | 219 | 160 | 100 | 15 |
| Neopterin | 1.6 | 254.1 | 206.1; 190.1 | 100 | 20 |
| Neopterin-13C5 | 1.7 | 259 | 210 | 120 | 10 |
| N-methylserotonin | 8.2 | 191 | 160 | 90 | 12 |
| Norepinephrine | 2.5 | 152 | 107 | 110 | 20 |
| Normetanephrine | 4.6 | 166 | 134 | 100 | 15 |
| Normetanephrine-d3 | 4.7 | 187.2 | 137.1 | 100 | 15 |
| Phenylalanine | 5.8 | 166 | 120 | 100 | 15 |
| Quinolinic acid | 1.9 | 168.1 | 106.1; 78.1 | 95 | 15; 30 |
| Quinolinic acid-d3 | 1.9 | 171.1 | 109.1; 81.1 | 95 | 15; 30 |
| Serotonin | 7.15 | 177; 160 | 160; 132; 105 | 90; 120 | 7; 20; 30 |
| Serotonin-d4 | 7.12 | 181.1 | 164; 136 | 90; 120 | 7; 20 |
| Tryptamine | 8.0 | 161.1 | 144.1;115.1 | 95 | 10; 30 |
| Tryptophol | 7.2 | 162.1 | 144.2 | 100 | 13 |
| Tyrosine | 3.7 | 182 | 165; 136 | 90 | 5; 10 |
| Vanilylmandelic acid | 3.5 | 197 | 138; 137 | 100 | 10; 24 |
| Xanthurenic acid | 6.7 | 206.1 | 160; 132.1 | 95 | 20; 30 |
| Xanthurenic-d4 acid | 6.8 | 210.1 | 164.1;136.1 | 95 | 20; 30 |

Table S6: MRM transitions for amino acid profiling

| Name | Retention time | Precursor ion | Product ion | Cone | CE |
| --- | --- | --- | --- | --- | --- |
| Gly | 1.21 | 76.0 | 30.0 | 17 | 10 |
| 13C215N-Gly | 1.21 | 79.0 | 32.0 | 17 | 10 |
| Ala | 1.24 | 90 | 44 | 18 | 10 |
| D4-Ala | 1.24 | 94 | 48 | 18 | 10 |
| Pro | 1.37 | 116.1 | 70.1 | 11 | 13 |
| Orn | 1.08 | 133.1 | 70.1 | 18 | 20 |
| D6-Orn | 1.08 | 139.1 | 76.1 | 18 | 20 |
| Leu | 2.94 | 132.1 | 86.1 | 17 | 10 |
| D3-Leu | 2.91 | 135.1 | 89.1 | 17 | 10 |
| ILe | 2.68 | 132.1 | 86.1 | 17 | 10 |
| Val | 1.5 | 118.1 | 72.1 | 19 | 13 |
| D8-Val | 1.5 | 126.1 | 80.1 | 19 | 13 |
| Asp | 1.26 | 134 | 116.1 | 20 | 13 |
| D3-Asp | 1.26 | 137 | 119.1 | 20 | 13 |
| Glu | 1.29 | 148.1 | 130 | 20 | 13 |
| D5-Glu | 1.29 | 153 | 135 | 20 | 13 |
| Met | 1.93 | 150.1 | 133 | 18 | 10 |
| D3-Met | 1.93 | 153.1 | 136 | 18 | 10 |
| Phe | 3.74 | 166.1 | 120.1 | 20 | 12 |
| D5-Phe | 3.74 | 171 | 125 | 20 | 12 |
| Arg | 1.11 | 175.1 | 70.1 | 22 | 21 |
| D7-Arg | 1.11 | 182 | 77 | 22 | 21 |
| Cit | 1.28 | 176.1 | 113.1 | 16 | 16 |
| D2-Cit |  | 178.1 | 115.1 | 16 | 16 |
| Tyr | 2.47 | 182.1 | 136.1 | 20 | 13 |
| D4-Tyr |  | 186 | 140 | 20 | 13 |
| Ser | 1.22 | 106 | 60 | 20 | 13 |
| Thr | 1.28 | 120 | 56 | 20 | 13 |
| Lys | 1.08 | 147 | 84 | 20 | 13 |
| Trp | 4.23 | 205 | 188 | 20 | 13 |
| His | 1.10 | 156 | 110 | 20 | 13 |

Table S7: MRM parameters used for acylcarnitine profiling

| Analyte | Abbreviation | Precursor ion | Product ion | CE |
| --- | --- | --- | --- | --- |
| Carnitine | C0 | 162 | 103 | 17 |
| Carnitine-D9 | C0-ISTD | 171 | 103 | 17 |
| Acetylcarnitine | C2 | 204 | 85 | 18 |
| Acetylcarnitine-D3 | C2-ISTD | 207 | 85 | 18 |
| Propionylcarnitine | C3 | 218 | 85 | 19 |
| Propionylcarnitine -D3 | C3-ISTD | 221 | 85 | 19 |
| Butyrylcarnitine | C4 | 232 | 85 | 20 |
| Butyrylcarnitine-D3 | C4-ISTD | 235 | 85 | 20 |
| Valerylcarnitine | C5 | 246 | 85 | 20 |
| Valerylcarnitine-D9 | C5-ISTD | 255 | 85 | 20 |
| Tiglylcarnitine | C5:1 | 244 | 85 | 20 |
|  | C5-DC | 276 | 85 | 20 |
| Hexanoylcarnitine | C6 | 260 | 85 | 22 |
| Hexanoylcarnitine-D3 | C6-ISTD | 263 | 85 | 22 |
| Octanoylcarnitine | C8 | 288 | 85 | 24 |
| Octanoylcarnitine -D3 | C8-ISTD | 291 | 85 | 24 |
| Octenoylcarnitine | C8:1 | 286 | 85 | 24 |
| Decanoylcarnitine | C10 | 316 | 85 | 26 |
| Decanoylcarnitine -D3 | C10-ISTD | 319 | 85 | 26 |
| Decenoylcarnitine | C10:1 | 314 | 85 | 26 |
| Decadienylcarnitine | C10:2 | 312 | 85 | 26 |
| Dodecanoylcarnitine | C12 | 344 | 85 | 27 |
| Dodecanoylcarnitine -D3 | C12-ISTD | 347 | 85 | 27 |
| Dodecenoylcarnitine | C12:1 | 342 | 85 | 27 |
| Tetradecadienylcarnitine | C14:2 | 368 | 85 | 27 |
| Hexadecanoylcarnitine | C16 | 400 | 85 | 28 |
| Hexadecanoylcarnitine -D3 | C16-ISTD | 403 | 85 | 28 |
| Octadecanoylcarnitine | C18 | 428 | 85 | 28 |
| Octadecanoylcarnitine -D3 | C18-ISTD | 431 | 85 | 28 |
| Octadecenoylcarnitine | C18:1 | 426 | 85 | 26 |
| Octadecadienylcarnitine | C18:2 | 424 | 85 | 20 |
| Hydroxyvalerylcarnitine | C5-OH | 262 | 85 | 26 |
| Hydroxyhexadecenoylcarnitine | C16:1-OH | 398 | 85 | 26 |
| Hydroxyoctadecenoylcarnitine | C18:1-OH | 442 | 85 | 28 |

Table S8: MRM parameters used for ADMA and SDMA profiling

| Name | Precursor ion | Product ion | Cone | CE |
| --- | --- | --- | --- | --- |
| Arginine | 175.1 | 70.1 | 22 | 21 |
| D7-Arg | 76.0 | 30.0 | 22 | 21 |
| ADMA | 203.2 | 46.1 | 22 | 21 |
| SDMA | 203.2 | 172.1 | 22 | 21 |
| Choline | 60.1 | 104.1 | 22 | 21 |

Table S9: VIP score of the metabolites

| **Metabolite** | **Vip score** |
| --- | --- |
| GSG ratio | 1,96203 |
| Glutamic acid | 1,77999 |
| Choline | 1,70533 |
| Tryptophol | 1,70136 |
| DMG | 1,62409 |
| C5 | 1,61634 |
| Vanillylmandelic acid | 1,60335 |
| Methionine sulfoxide | 1,54399 |
| C10-2 | 1,50759 |
| N-MeArg | 1,48951 |
| C18-OH | 1,45482 |
| Leucine | 1,41511 |
| Methione | 1,39062 |
| Kynurenine | 1,37788 |
| Valine | 1,36841 |
| Kyn/Trp ratio | 1,36513 |
| 3-Amino IsoButyric acid | 1,35403 |
| C14-OH | 1,31072 |
| Kynurenic Acid | 1,31061 |
| C16-OH | 1,26914 |
| Citrulline | 1,25999 |
| Xanthurenic acid | 1,24793 |
| C10-1 | 1,22469 |
| Phenylalanine | 1,21686 |
| Fischer ratio | 1,18299 |
| Anthranillic acid | 1,1814 |
| 3-OHKynurenine | 1,17948 |
| C0 | 1,15743 |
| C18 | 1,15643 |
| SDMA | 1,15576 |
| C16 | 1,15022 |
| Glycine | 1,11157 |
| Methionine | 1,09881 |
| Isoleucine | 1,07305 |
| C18-1-OH | 1,05068 |
| Neopterin | 1,03984 |
| HIAA | 0,9983 |
| Indole-3-Carboxamide | 0,996071 |
| Aspartic acid | 0,995019 |
| Proline | 0,979976 |
| C16-1-OH | 0,956932 |
| Norepinephirne | 0,931444 |
| C18-1 | 0,901122 |
| Serine | 0,900757 |
| Threonine | 0,898934 |
| C16-1 | 0,894824 |
| Alanine | 0,889206 |
| ADMA | 0,879485 |
| C5-OH | 0,862768 |
| C10 | 0,86037 |
| C14 | 0,84512 |
| Betaine | 0,840447 |
| Methionine+MetSO4 | 0,837243 |
| AOR ratio | 0,811616 |
| C3 | 0,804621 |
| C18-2 | 0,801391 |
| Ornithine | 0,7763 |
| Tyrosine | 0,75595 |
| C5-1 | 0,75323 |
| TMAO | 0,722061 |
| 3-OHAnthranillic acid | 0,70753 |
| C5-DC | 0,703989 |
| GABA | 0,68758 |
| C8-1 | 0,630087 |
| Quinolinic acid | 0,625877 |
| GABR ratio | 0,616424 |
| Acetylcholine | 0,604656 |
| Normetanephrine | 0,602259 |
| Cystathionine | 0,590664 |
| C12 | 0,585908 |
| 5-OH-tryptophan | 0,582008 |
| Indol-3-Propionic acid | 0,574803 |
| C12-1 | 0,551841 |
| Histidine | 0,550582 |
| Serotonin | 0,510994 |
| Indol-3-Lactic acid | 0,507602 |
| Melatonin | 0,486364 |
| Asparagine | 0,445844 |
| C14-1 | 0,445456 |
| Indol-3-Acetic acid | 0,432547 |
| C6 | 0,403926 |
| Tryptophan | 0,398511 |
| Arginine | 0,393156 |
| Biopterin | 0,380597 |
| Indole-3-Butyric acid | 0,35988 |
| C6-DC | 0,346646 |
| C14-2 | 0,336654 |
| C8 | 0,330771 |
| Arginine | 0,309203 |
| C4 | 0,296436 |
| Indol-acrylic acid | 0,281265 |
| Metanephrine | 0,256904 |
| C2 | 0,229321 |
| Lysine | 0,0517287 |

Table S10. Characteristics of the correlation networks of the considered groups of patients

|  | Non-CVD | IHD | AMI |
| --- | --- | --- | --- |
| Number of nodes | 73 | 67 | 65 |
| Number of edges | 74 | 86 | 77 |
| Avg. number of neighbors | 2.32 | 2.93 | 3.92 |
| Network density | 0.047 | 0.055 | 0.17 |
| Network heterogeneity | 0.435 | 0.521 | 0.442 |
| Network centralization | 0.036 | 0.06 | 0.146 |
| Connected components | 8 | 7 | 12 |

Figure S1. PCA analysis of the analyzed samples

*
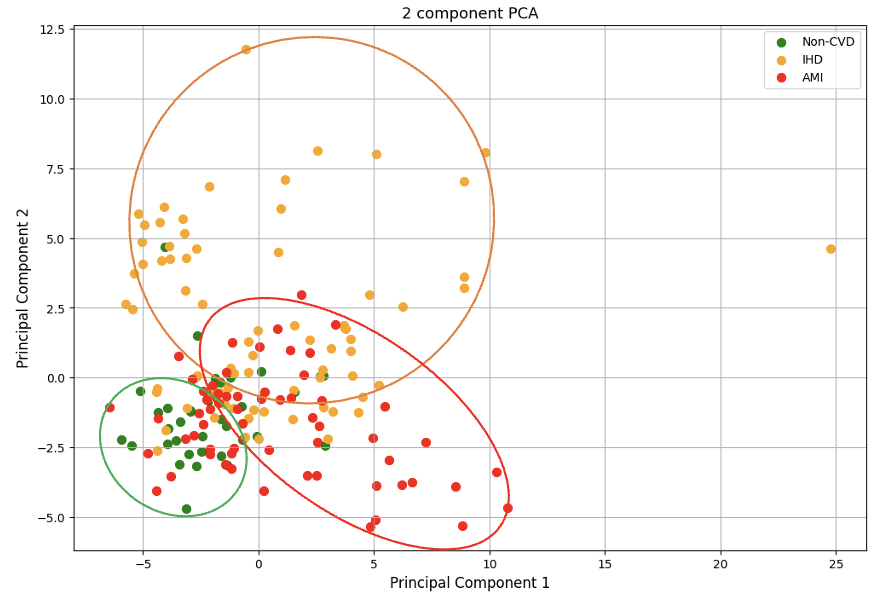
*

Figure S2. OPLS-DA analysis


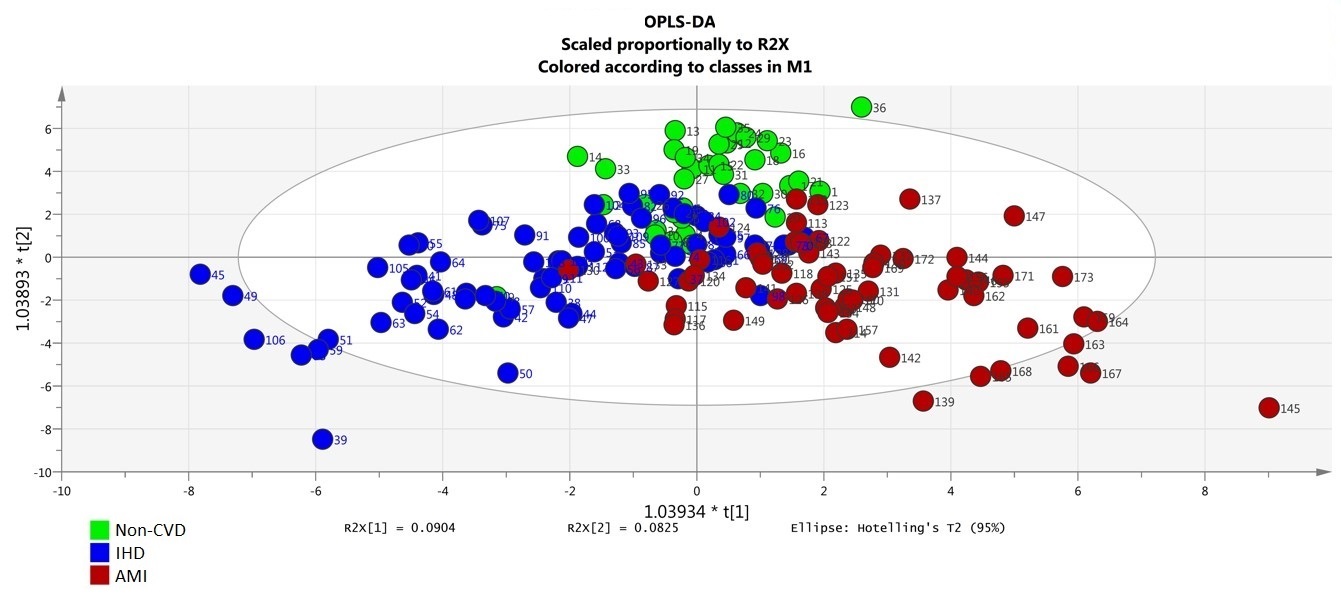

Supplement: Supplementary file 1 — Supplementary Material 1 [file 41598_2024_75635_MOESM1_ESM.docx]
